# Optogenetic manipulation of cardiac electrical dynamics using sub-threshold illumination: dissecting the role of cardiac alternans in terminating rapid rhythms

**DOI:** 10.1007/s00395-022-00933-8

**Published:** 2022-04-29

**Authors:** V. Biasci, L. Santini, G. A. Marchal, S. Hussaini, C. Ferrantini, R. Coppini, L. M. Loew, S. Luther, M. Campione, C. Poggesi, F. S. Pavone, E. Cerbai, G. Bub, L. Sacconi

**Affiliations:** 1 European Laboratory for Non-Linear Spectroscopy - LENS, Sesto Fiorentino, Italy; 2 Department of Experimental and Clinical Medicine, University of Florence, Florence, Italy; 3 Department of Neurology, Psychology, Drug Sciences and Child Health, University of Florence, Florence, Italy; 4 National Institute of Optics (INO-CNR), Sesto Fiorentino, Italy; 5 Max Planck Institute for Dynamics and Self-Organization, Gottingen, Germany; 6 Center for Cell Analysis and Modeling, University of Connecticut, Farmington, CT USA; 7 Institute of Neuroscience (IN-CNR) and Department of Biomedical Science, University of Padua, Padua, Italy; 8 Department of Physics and Astronomy, University of Florence, Sesto Fiorentino, Italy; 9 Department of Physiology, McGill University, Montreal, Canada; 10 Institute for Experimental Cardiovascular Medicine, University Heart Center and Medical Faculty, University of Freiburg, Freiburg, Germany

**Keywords:** Cardiac alternans, Ventricular tachycardias, Voltage imaging, Optogenetics

## Abstract

Cardiac action potential (AP) shape and propagation are regulated by several key dynamic factors such as ion channel recovery and intracellular Ca^2+^ cycling. Experimental methods for manipulating AP electrical dynamics commonly use ion channel inhibitors that lack spatial and temporal specificity. In this work, we propose an approach based on optogenetics to manipulate cardiac electrical activity employing a light-modulated depolarizing current with intensities that are too low to elicit APs (sub-threshold illumination), but are sufficient to fine-tune AP electrical dynamics. We investigated the effects of sub-threshold illumination in isolated cardiomyocytes and whole hearts by using transgenic mice constitutively expressing a light-gated ion channel (channelrhodopsin-2, ChR2). We find that ChR2-mediated depolarizing current prolongs APs and reduces conduction velocity (CV) in a space-selective and reversible manner. Sub-threshold manipulation also affects the dynamics of cardiac electrical activity, increasing the magnitude of cardiac alternans. We used an optical system that uses real-time feedback control to generate re-entrant circuits with user-defined cycle lengths to explore the role of cardiac alternans in spontaneous termination of ventricular tachycardias (VTs). We demonstrate that VT stability significantly decreases during sub-threshold illumination primarily due to an increase in the amplitude of electrical oscillations, which implies that cardiac alternans may be beneficial in the context of self-termination of VT.

**Supplementary Information:**

The online version contains supplementary material available at 10.1007/s00395-022-00933-8

## Introduction

Cardiac action potential (AP) onset and propagation across the heart are physiological processes regulated by several key dynamic factors such as collective ion channel recovery as well as intracellular Ca^2+^-cycling [34, 35]. Pathological conditions alter these properties, often resulting in beat-to-beat changes of AP properties, a phenomenon called “alternans” [42], which is linked to an increased risk of life-threatening re-entrant cardiac arrhythmias. Experimental methods for manipulating AP dynamics are limited: pharmacological approaches [18, 19, 41] lack spatial and temporal specificity and in some cases are only partially reversible. In this work, we propose an approach that uses optogenetics to manipulate cardiac AP dynamics, enabling the exploration of the role that cardiac alternans plays in sustaining or terminating re-entrant cardiac arrhythmias.

Optogenetics combines the use of light-sensitive proteins, genetically expressed in cells/tissues of interest, with high-resolution optical tools for contactless control and monitoring of electrical activity in excitable cells. Expression of light-sensitive depolarizing ion channels, such as channelrhodopsin-2 (ChR2) in excitable cells, enables the optical induction of APs [10, 26]. Following the tremendous advances achieved in neuroscience, optogenetics has been successfully extended to cardiac research [13]. Optogenetics-based strategies have been proposed as alternatives to wired electrical stimulation for cardiac pacing [6, 30] and cardioversion [4, 5, 9, 11, 16, 31, 36, 38]. Moreover, optogenetics recently emerged as a robust tool for investigating wave dynamics in cardiac tissue, studying the mechanisms underlying the induction, maintenance and control of cardiac arrhythmias [7, 12, 14, 40].

Importantly, optogenetic interventions have so far been mostly used for generating transient and intense depolarizing currents for APs triggering or cardioversion. However, the utility of ChR2 for imposing a continuous depolarizing current with amplitudes that are too low to elicit APs (sub-threshold illumination), but are sufficient to fine-tune AP electrical dynamics has not been fully investigated. Recent studies performed primarily *in silico* revealed novel insights about the use of sub-threshold illumination to destabilize and terminate spiral waves in a two-dimensional (2D) model of adult mouse ventricle [20]. Moreover, sub-threshold stimulation has been used to manipulate the shape of cardiac APs in human atrial models at different spatial scales [21]. In our present work, we explore the effects of sub-threshold illumination both in ex vivo and *in silico* experiments. We first characterized the electrophysiological response to sub-threshold illumination of single cardiomyocytes isolated from mice expressing ChR2 (under the control of a cardiac-specific α-myosin heavy chain promoter) using patch clamp techniques. Next, using an optical mapping system operating in the near-infrared range, in combination with a stimulator generating customisable patterns of blue light, we characterized AP properties in isolated mouse hearts expressing ChR2. We found that sub-threshold illumination alters the shape of APs, allowing us to create spatiotemporal heterogeneities in AP properties across the heart in a completely reversible manner. More importantly, we also found that sub-threshold illumination promotes changes in the dynamics of cardiac electrical activity: sub-threshold illumination increases cardiac alternans, which we studied in the context of self-termination of ventricular tachycardia (VT).

## Materials and methods

### Mouse model generation

Transgenic mice (ChR2-mhc6-cre +) with cardiomyocyte-specific expression of ChR2 (H134R variant) and control (CTRL) mice (ChR2-wtwt-cre +) were generated [46] and employed in this study. All animal handling and procedures were performed in accordance with the guidelines from Directive 2010/63/EU of the European Parliament on the protection of animals used for scientific purposes. The experimental protocol was approved by the Italian Ministry of Health (protocol number 944/2018-PR).

### Cell isolation and patch clamp recording

Ventricular cardiomyocytes from CTRL and ChR2 mice were isolated by enzymatic dissociation as previously described [39]. Briefly, mice (6 months old) were heparinized (0.1 mL at 5000 units/mL) and anesthetized by inhaled isoflurane (5%). The excised heart was immediately bathed in cell isolation buffer and the proximal aorta was cannulated for perfusion in a Langendorff system. The buffer solution contained (in mM): 120 NaCl, 1.2 MgCl_2_, 10 KCl, 1.2 KH_2_PO_4_, 10 glucose, 10 HEPES, 20 taurine, 5 pyruvate, pH 7.4 (adjusted with NaOH), oxygenated with oxygen. After perfusion at 36 °C for 15 min with a constant flow of 3 mL/min, the solution was then switched to a recirculating enzyme solution, made from the same buffer with the addition of 0.1 mg/mL Liberase^™^ (Roche Applied Sciences, Penzberg, Germany). After 8 min, the ventricles were excised and cut into small pieces in buffer solution added with 1 mg/mL of bovine serum albumin (BSA). Gentle stirring was used to further facilitate dissociation of myocytes. The cell suspension was left to settle, and the cell pellet was resuspended in Tyrode buffer, containing (in mM): 133 NaCl, 4.8 KCl, 1.2 MgCl_2_, 10 glucose and 10 HEPES, pH 7.4 (adjusted with NaOH). The calcium concentration of the cell suspension was gradually increased to 0.6 mM by adding 15 μL of CaCl_2_ (0.1 M). Finally, cardiomyocytes were superfused with Tyrode buffer containing 1.8 mM CaCl_2_ during patch-clamp experiments. Patch-clamp data recordings and analysis were performed as previously described [8] using a Multiclamp700B amplifier in conjunction with pClamp10.0 and a DigiData 1440A AD/DA interface (Molecular Devices, San Jose, CA, USA). For resting membrane potential (V_rest_) and AP recordings, the pipette solution contained (in mM): 130 potassium aspartate, 0.1 Na-GTP, 5 Na_2_-AT, 11 EGTA, 5 CaCl_2_, 2 MgCl_2_, 10 HEPES (pH 7.2 with KOH). Intracellular access was obtained via whole-cell ruptured patch. All experiments were performed at 36 ± 0.5 °C. APs were electrically elicited by inward current injection (3 ms current square pulses, 500–1000 pA) at a stimulation frequency of 1 Hz. To assess cell excitability, we increased the inward current pulse (3 ms duration) gradually (50 pA per step) until an AP was induced. Membrane resistance (*R*_m_) was measured in the voltage clamp mode (− 80 mV) applying a double step of ± 10 mV. For sub-threshold ChR2 activation, the cells were globally illuminated using a light emitting diode (LED) operating at a wavelength centered at 470 nm (SPECTRA X light engine, Lumencor, Beaverton, OR, USA) and a × 20 objective (NA; 0.5, HCX PL FLUOTAR, Leica Microsystems, Wetzlar, Germany). Light intensities (LIs) were measured at the sample site using a photodiode sensor (PD300-3 W, Ophir Optronics, Jerusalem, Israel).

### Isolated and perfused mouse hearts

The excised heart was immediately bathed in Krebs–Henseleit (KH) solution and cannulated through the aorta. The KH buffer contained (in mM): 120 NaCl, 5 KCl, 2 MgS_2_ O_4_—7H_2_O, 20 NaHCO_3_, 1.2 NaH_2_PO_4_—H_2_O, 1.8 CaCl_2_ and 10 glucose, pH 7.4 when equilibrated with carbogen. Cardiac contraction was inhibited during the entire experiment with 5 μM blebbistatin (Enzo Life Sciences, Farmingdale, NY, USA) in the solution. The cannulated heart was perfused through the aorta (using a horizontal-Langendorff perfusion system) with the KH solution and then transferred to a custom-built optical mapping chamber at a constant flow of 2.5 mL/min at (36 ± 0.5) °C. Two platinum electrodes were placed below the heart for monitoring cardiac electrical activity via electrocardiogram (ECG). 1 mL of perfusion solution containing the voltage sensitive dye (VSD; di-4-ANBDQPQ, 6 μg/mL, University of Connecticut Health Center, Farmington, CT, USA) [24] was bolus injected into the aorta. All the experiments were performed at (36 ± 0.5) °C within 1 h after dye loading to avoid potential re-distribution of the dye and accumulation of phototoxic by-products.

### All-optical imaging and manipulation platform

Optical mapping and control were performed using a custom-made mesoscope. The whole mouse heart was illuminated in a wide-field configuration using a 2 × objective (TL2x-SAP, Thorlabs, Newton, NJ, USA) and a LED operating at a wavelength centered at 625 nm (M625L3, Thorlabs, Newton, NJ, USA), followed by an excitation band-pass filter at 640/40 nm (FF01- 640/40–25, Semrock, Rochester, NY, USA). The heart was illuminated with a maximum intensity of 1 mW/mm^2^. A dichroic beam splitter (FF685- Di02-25 × 36, Semrock) followed by a band-pass filter at 775/140 nm (FF01-775/140–25, Semrock) was used for collecting the VSD-emitted fluorescent signal. A × 20 objective (LD Plan-Neofluar × 20/0.4 M27, Carl Zeiss Microscopy, Oberkochen, Germany) was used to focus the fluorescent signal on a central portion (128 × 128 pixels) of the sensor of a sCMOS camera (OrcaFLASH 4.0, Hamamatsu Photonics, Shizuoka, Japan) operating at a frame rate of 1 kHz (1 ms actual exposure time). The detection path allows a field of view (at the object space) of 10.1 × 10.1 mm^2^ sampled with a pixel size of 80 μm. To manipulate cardiac electrical activity, a Lightcrafter 4500 projector (Texas Instruments, Dallas, TX, USA), operating at a wavelength of 470 nm, was used for projection of user-defined light patterns onto the heart surface. LIs were measured at sample site using a photodiode sensor (PD300-3 W, Ophir Optronics, Jerusalem, Israel). The system was used to optically probe AP propagation in mouse hearts during sub-threshold illumination using user-defined illumination patterns (whole heart, right half, and left half of the heart). Hearts were electrically paced at the apex with a bipolar electrode using an isolated constant voltage stimulator (DS2A, Digitimer, Welwyn Garden City, Hertfordshire, UK). As described before [17], the optical platform was implemented with a custom LabVIEW software program (LabVIEW 2015, Version 15.0 64-bit, National Instruments, Austin, TX, USA), allowing it to mimic re-entrant VT during sub-threshold optogenetic illumination. Briefly, the apex of the heart was electrically paced, and the induced excitation wave propagated toward the base of the heart. Once an AP was optically detected in a region of interest (ROI) placed at the base of the heart, a new trigger was generated at the apex after a user-defined fixed delay, thus restarting the cycle. For each delay time (DT), re-entrant VT was established for 10 s.

### Data and image analysis

All programs for data acquisition and analysis were developed with LabVIEW (National Instruments). For optical recordings, ΔF/F0 imaging of cardiac electrical activity was performed by processing raw data: for each frame, the mean baseline was subtracted, and the frame was subsequently normalized to the mean baseline, yielding a percentage change in fluorescence over time. For each heart, AP kinetics parameters were measured, trace by trace, to get the mean values after averaging five to ten subsequent trials. AP maximum rising slope (APRS), AP duration (APD) at 50% of repolarization (APD50), APD at 70% of repolarization (APD70), and APD at 90% of repolarization (APD90) were measured in a selected region of interest (ROI) of 10 × 10 pixels (≈ 1 mm^2^). APD was determined relative to the time of maximum depolarization. During slow pacing, APDs were measured considering the diastolic potential preceding the beat, while during fast pacing bursts and VTs (where a full repolarization could not be measured), AP parameters were measured relative to fluorescence baseline before and after the stimulation burst. In VTs, conduction time (CT) was calculated as cycle length (CL)-DT. APRS, APD50, APD70 and CT alternans were calculated using the following formula: $(\sum_{i=1}^{n-1}\left|X\text{i+1}-X{\text{i}}\right|)/(n-$ 1), where X is the parameter of interest. APRS and APD90 were also analyzed across the whole ventricle after a spatial binning of 4 × 4 pixels, generating maps containing APRS and APD90 values across the ventricle. In addition, spatial dispersion of these parameters were assessed by calculating the standard deviation (SD) of values across all pixels. Conduction velocity (CV) was calculated after a spatial binning of 4 × 4 pixels using a multi-vector approach: a seed reference pixel was arbitrarily chosen, and the cross-correlation of the fluorescence trace was calculated pixel by pixel, to estimate the temporal shift among every pixel (activation map). Next, local velocity maps were generated by calculating the delay between adjacent pixels divided by the pixel size. Since the local direction of AP wave-front is represented by a vector for each pixel, the mean CV was calculated by averaging local CVs. Graphic representation of data was obtained using OriginPro 2018, version 9.5 64-bit (OriginLab Corporation, Northampton, MA USA).

### Numerical study

The effect of sub-threshold illumination was also investigated using an ionic mathematical model of the optogenetically modified adult mouse ventricular monolayer. Electrical activity in a single cardiac cell is modeled according to Eq. 1:

1 $$dV/dt \, = \, - \, \left( {I_{ion} + \, I_{stim} } \right)/C_{m} ,$$

where *V* is the transmembrane voltage that arises from ionic gradients that develop across the cell membranes, *C*_m_ is the membrane capacitance of each cell and *I*_stim_ is the electrical stimulation current. The total ionic current *I*_ion_ flowing across the membrane of a single cell is mathematically described using the electrophysiological model of an adult mouse ventricular cardiomyocyte, introduced by Bondarenko, including the model improvements in Petkova-Kirova [3, 32]. The 15 total currents flowing through the cell membrane are: the fast Na^+^ current (*I*_Na_), the L-type Ca^2+^ current (*I*_Ca,L_), the Ca^2+^ pump current (*I*_pCa_), the rapidly recovering transient outward K^+^ current (*I*_to,f_), the slowly recovering transient outward K^+^ current (*I*_to,s_), the rapid delayed rectifier K^+^ current (*I*_Kr_), the ultrarapid delayed rectifier K^+^ current (*I*_Kur_), the non-inactivating steady-state voltage-activated K^+^ current (*I*_ss_), the time-independent inwardly rectifying K^+^ current (*I*_K1_), the slow delayed rectifier K^+^ current (I_Ks_), the Na^+^/Ca^2+^ exchange current (*I*_Na/Ca_), the Na^+^/K^+^ pump current (*I*_Na/K_), the Ca^2+^ activated Cl^−^ current (*I*_Cl(Ca)_), the background Ca^2+^ current (*I*_Ca,b_) and the background Na^+^ current (*I*_Na,b_).

In a ventricular monolayer (2-dimentional (2D) domain), cardiac cells communicate with each other through intercellular coupling. The membrane voltage is then modeled using a reaction–diffusion equation (Eq. 2):

2 $$dV/dt \, = \nabla .(D\nabla V) \, - \, \left( {I_{ion} + \, I_{stim} } \right)/C_{m} ,$$

The first term on the right-hand side of the equation controls the intercellular coupling. D is the diffusion tensor, assumed here to be a scalar and set to the value 0.0014 cm^2^/ms to obtain isotropic plane wave propagation with a velocity of 42 cm/s. In this 2D simulation domain, spatial and temporal resolution are considered with values of 0.025 cm and 10^–4^ ms, respectively. We also applied a no-flux boundary condition at the unexcitable borders of this 2D region.

To make this model light responsive, we added the mathematical model of channelrhodopsin-2 (ChR2) [44] to this ionic cell model of ventricular mouse heart. This photo-cycle model describes the dynamics of a non-selective cation channel ChR2 that responds to blue light with a wavelength of 470 nm. The inward ChR2 current (I_ChR2_) is mathematically described by the following equation (Eq. 3):

3 $$I_{chR2} = \, g_{ChR2} G\left( V \right)\left( {O_{1} + \, \gamma O_{2} } \right)\left( {V \, - \, E_{ChR2} } \right).$$

Here, *g*_ChR2_ is the conductance with value of 0.4 mS/cm^2^, G(V) is the voltage rectification function, O_1_ and O_2_ are the open state probabilities of the ChR2, γ is the ratio of conductance of O_2_/O_1_ with value of 0.1, and E_ChR2_ is the reversal potential of this channel with value of 0 mV. The detailed description and values of other parameters can be found in [44]. By including the mathematical model of ChR2 kinetics in the monolayer model of ventricular mouse heart, it is possible to simulate the effects of light on the ChR2 expressing monolayer at the 2D mono-domain level. To investigate the effect of sub-threshold illumination on the velocity of a propagating planar wave, a 2D domain with size of 2.5 × 2.5 cm^2^ was continuously illuminated globally at different LIs (0, 0.005, 0.010, 0.0153, 0.020, 0.025, 0.030 mW/mm^2^). Then we measured the CV of the planar wave by measuring the time the wave travels through two spatially distinct points with coordinates of *X*: 0.625 cm, *Y*: 1.25 cm and *X*: 1.875 cm, *Y*: 1.25 cm. Global and structured illumination patterns were used to study the morphology of an excitation wave AP under sub-threshold illumination. In both cases, planar waves were triggered by a sequence of electrical pulses with a strength of 80 pA/pF, a pulse length of 0.5 ms, and a stimulation frequency of 5 Hz on the left side of the domain. Then, for the case of a global illumination pattern, we measured the APD90 and APA for an ROI selected in the center of the domain with a coordinate of (*X*: 1.25 cm, *Y*: 1.25 cm) while the planar wave passes through this single point. To visualize the difference in the CV of a planar wave propagating in illuminated and non-illuminated regions, we used a structured illumination pattern. To do this, we illuminated half of the area where the planar wave propagates perpendicular to the intersection of illuminated and non-illuminated regions.

### Statistics

For each experimental condition, data from one cell (in patch clamp measurements) or one heart (optical mapping measurements) was averaged, and this average was used for comparison and statistical analysis. Two-way repeated measures (RM) analysis of variance (ANOVA) tests were used to compare electrophysiological features between CTRL and ChR2 mice at different LIs. This method not only assessed the main effect of each categorical independent variable but also determines if there is any interaction between them, since the effects on the outcome of the change in one factor may depend on the magnitude of the other factor. For the comparison of means at specific LIs, the Tukey’s post hoc analysis was used. To investigate the general influence of illumination on AP features in CTRL and ChR2 mice, a regression test was applied: an ANOVA test was used to assess if the fitting function (linear or exponential) is significantly better than a constant function. In addition, the unpaired Student’s *t* test was used to compare two experimental groups, without another variable. A *p* value of < 0.05 was considered as indicative of a statistically significant difference between means (NS: *p* > 0.05; *p < 0.05; **p < 0.01, ***p < 0.001, ****p < 0.0001). Statistical analysis was performed using OriginPro 2018, version 9.5 64-bit and GraphPad Prism, version 8.4.3.

## Results

### Sub-threshold illumination in isolated cardiomyocytes

**Fig. 1:**
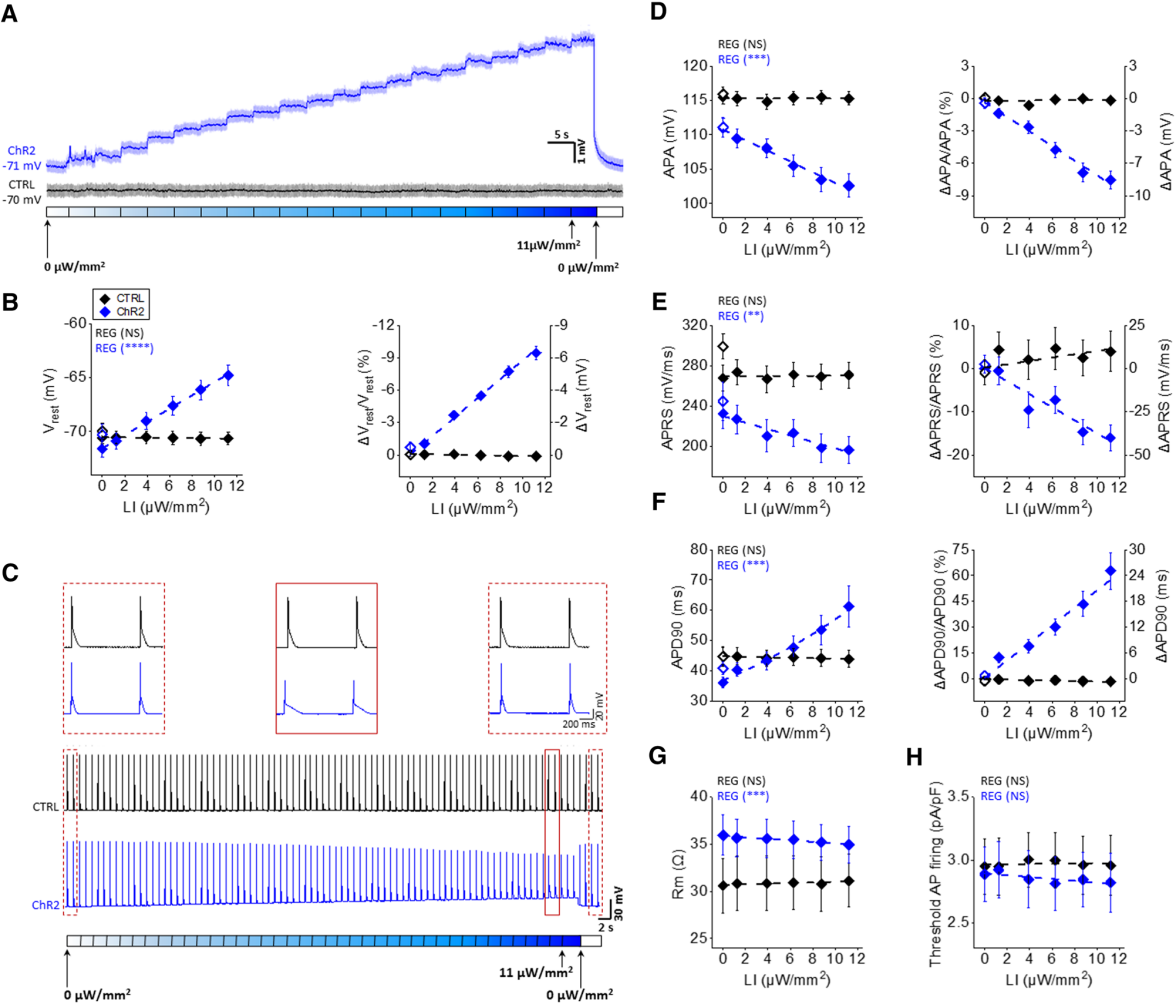
Sub-threshold illumination in single cardiomyocytes isolated from CTRL and ChR2 mouse hearts. **A** Representative traces of V_rest_ recorded in CTRL (black) and ChR2 cardiomyocytes (blue) during global and constant sub-threshold illumination of cardiomyocytes at increasing light intensities (LIs). The bottom bar shows the LIs used. Light was incremented of 2 µW/mm^2^ every 5 s in the range 0–11 µW/mm^2^. Blue light was turned off at the end of the illumination protocol to assess reversibility. B) Absolute values (left), percentage variation and absolute variation (right) of V_rest_ in CTRL and ChR2 cardiomyocytes. **C** Representative traces of APs recorded in CTRL (black) and ChR2 cardiomyocytes (blue) during global and constant sub-threshold illumination of the cell at several increasing LIs. APs were induced by current injection at a frequency of 1 Hz. The bottom bar shows the LIs used. Light was incremented in 2 µW/mm^2^ steps every 2 s in the range 0–11 µW/mm^2^ and then turned off at the end of the illumination protocol to assess reversibility. The top three panels show two successive APs recorded before (left dashed red frame), during (middle solid red frame) and after (right dashed red frame) sub-threshold illumination (zoom of traces in the bottom). **D**–**F** Absolute values (left), percentage variation and absolute variation (right) of APA, APRS and APD90 in CTRL and ChR2 cardiomyocytes. Empty diamonds represent values when the light was turned off at the end of the illumination protocol to assess reversibility. **G** Absolute values of membrane resistance (*R*_m_) in CTRL and ChR2 cardiomyocytes measured at diastolic potential. **H** Absolute values of current intensity normalized to cell capacitance required to achieve AP firing in CTRL and ChR2 cardiomyocytes. Data was collected from 6 CTRL mice (36 cardiomyocytes) and 10 ChR2 mice (30 cardiomyocytes). Data are reported as mean ± standard error of the mean (SEM) and a linear fit on experimental data was superimposed. Regression analysis results (REG; ANOVA test) are shown for both CTRL and ChR2 cardiomyocytes. No significant difference was found between CTRL and ChR2 cardiomyocytes in *V*_rest_, APA, APRS and APD in absence of sub-threshold illumination (two-way RM ANOVA test with Tukey’s post hoc test)

We first investigated the electrophysiological effects of sub-threshold illumination at the single cell level. Membrane potential was monitored in CTRL and ChR2 cardiomyocytes using patch clamp (current clamp configuration). V_rest_ of cardiomyocytes was recorded during global and constant sub-threshold illumination of cells with increasing LIs from 0 to 11 µW/mm^2^. As shown in the representative traces in Fig. 1A, V_rest_ in CTRL cardiomyocytes which do not express ChR2 is not affected by the blue light and remains constant during the entire measurement. On the other hand, ChR2 expressing cardiomyocytes (in blue) displayed an increase in V_rest_ as a function of irradiance, with a mean ΔV_rest_ of (7.47 ± 0.75) mV at the maximum irradiance (Fig. 1B). This behavior was expected, considering that ChR2 activation gives rise to an inward flux of cations (*I*_ChR2_), which depolarizes the cell. Interestingly, when the *V*_rest_ of the ChR2 cardiomyocytes reached more positive values following the light stimulus, an equilibrium point was immediately established (within the sampling resolution time), until a new level of irradiance was applied. Importantly, when the light was switched off at the end of the illumination protocol, the *V*_rest_ of the initial condition was immediately restored, demonstrating that the light-induced depolarization is fully reversible.

We then investigated the effects of sub-threshold illumination in presence of APs induced by inward current injection (3 ms current square pulses, 500–1000 pA, at 1 Hz frequency). Figure 1C shows representative traces of APs recorded in CTRL and in ChR2 cardiomyocytes during sub-threshold illumination with increasing LIs. These traces clearly show an increase in diastolic cell depolarization with increasing irradiance, as well as an altered AP shape in ChR2 cardiomyocytes; such changes were fully reversed when the light was turned off, and did not occur in CTRL cells. Specifically, we found a significant reduction of AP amplitude (APA), which decreased linearly with each increase of LI (Fig. 1D). Moreover, we observed changes in depolarization and repolarization times, which modified AP shape in ChR2 cardiomyocytes: AP rising slope (APRS) was significantly reduced (Fig. 1E)*,* while AP duration (APD) was significantly prolonged (Fig. 1F), by amounts that linearly depended on the intensity of blue light. In addition, a mild LI-dependent reduction in R_m_ was observed in ChR2 cardiomyocytes at diastolic potential (Fig. 1G), while no effect on the cellular excitability was detected (Fig. 1H). Finally, a light-mediated increase in refractoriness was found in ChR2 cardiomyocytes employing S1-S2 protocol (Supplemental Fig. S1). These findings demonstrate that constant sub-threshold illumination allows for a wide range of electrophysiological feature manipulations in single cardiomyocytes. Moreover, we explored the possibility to manipulate the AP in a time-selective manner. As show in Supplementary Fig. S2, by applying a sub-threshold light pulse only during the AP repolarization phase, we demonstrated the possibility to selectively increase APD90, while the other electrophysiological parameters (*V*_rest_, APA, APRS) remained unaffected.

### Sub-threshold illumination in intact hearts

**Fig. 2:**
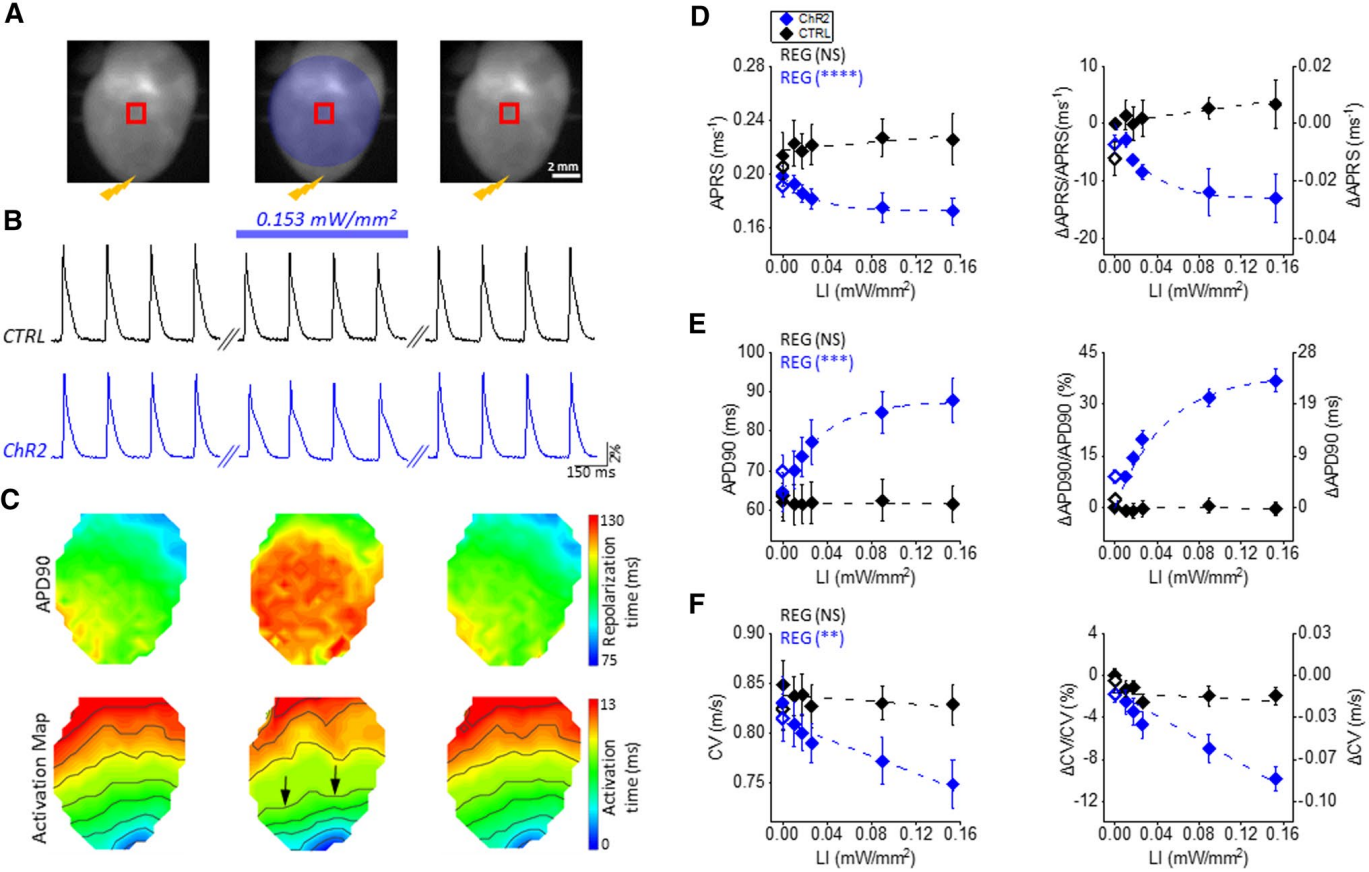
Sub-threshold illumination in intact hearts isolated from CTRL and ChR2 mice. **A** Representative fluorescence images of a mouse heart showing the illumination protocol. Mouse hearts were electrically paced at the apex using an electrode with a burst of 15 stimuli at 5 Hz (the location of electrical stimuli are shown using a yellow bolt symbol). At the same time the entire surface of the heart was constantly sub-threshold illuminated with increasing LIs. Electrical activity was optically recorded before (left panel), during (middle panel) and at the end (right panel) of sub-threshold illumination of the whole heart (filled blue circle). **B** Fluorescent signals (ΔF/F) extracted from the red ROIs in CTRL (black trace) and ChR2 (blue trace) mouse hearts. The blue bar shows the timing of the sub-threshold illumination with LI = 0.153 mW/mm^2^. **C** APD90 map (top) and activation map (bottom) of ChR2 mouse heart shown in A (LI = 0.153 mW/mm^2^). Black arrows highlight the light-mediated delay in AP wavefront propagation. **D**–**F** Absolute values (left), percentage variation and absolute variation (right) of APRS, APD90 and CV in CTRL and ChR2 hearts. Empty diamonds represent values when the light was turned off at the end of the illumination protocol to assess reversibility. Data were collected from 7 CTRL and 7 ChR2 hearts. Data is reported as mean ± SEM and exponential (in **D** and **E**) and linear (in **F**) fit on experimental data was superimposed. Regression analysis results (REG; ANOVA test) are shown for both CTRL and ChR2 hearts. No significant difference was found between CTRL and ChR2 hearts in APRS, APD and CV in the absence of sub-threshold illumination (two-way RM ANOVA test with Tukey’s post hoc test)

To assess the possibility of manipulating electrophysiological characteristics in whole hearts, we measured the effects of sub-threshold illumination in intact Langendorff-perfused mouse hearts, stained with a red-shifted VSD using the mesoscopic imaging platform. To overdrive the sinus rhythm (SR; 5.1 ± 0.2 Hz in CTRL (*n* = 7) and 4.5 ± 0.2 Hz in ChR2 (*n* = 17); *t* test: NS) CTRL and ChR2 mouse hearts were electrically paced at the apex with a burst of 15 stimuli at 5 Hz. During this pacing protocol, the entire heart surface was constantly sub-threshold illuminated with blue light at different LIs (Fig. 2A). Importantly, the use of a VSD excitation wavelength of 640 nm did not induce cell depolarization in the ChR2 mouse heart [40] allowing us to assess the effect of sub-threshold illumination employing optical mapping. However, the blue light used for sub-threshold illumination (470 nm) also excites the VSD, causing an increase of the fluorescence baseline signal (Supplementary Fig. S3A). This crosstalk precludes the possibility of manipulating AP in a time-selective manner and, more significantly, also affects the quantification of APA during constant sub-threshold illumination (Supplementary Fig. S3B). This is because the 470 nm absorption occurs in a spectral region in which the VSD is less sensitive to the membrane potential, thus producing an overall reduction of the actual VSD sensitivity.

Representative optical recordings (Fig. 2B) show that AP shape is manipulated by sub-threshold illumination (LI 0.153 mW/mm^2^) in ChR2 mouse hearts. Normal AP shape is restored when the light is turned off, and no effects of sub-threshold illumination were observed in CTRL mouse hearts. Notably, APD90 and activation maps (Fig. 2C) reveal a marked increase in APD and a delay in AP wavefront propagation within the illuminated area in ChR2 hearts. In line with patch-clamp experiments, APRS decreased (Fig. 2D), and APD90 prolonged (Fig. 2E) with increasing LIs. The light-mediated effects on APRS and APD90 occurred uniformly across the illuminated region without increasing AP spatial dispersion (supplementary figure S4). Finally, an inverse linear relationship between LI and CV was found (Fig. 2F) in ChR2 hearts.

Since light scattering within the tissue could create heterogeneities which affect our experimental results, we assessed whether our observations in whole hearts were consistent with homogeneous illumination. Specifically, we tested if our experimental findings could be computationally predicted by using a mouse ventricular monolayer mathematical model, where a homogenous sub-threshold illumination was imposed (Supplemental Fig. S5A, B). Consistent with our experimental findings, the model predicted APD prolongation as well as APA and CV reduction as a function of LI (Supplemental Fig. S5C–E).

**Fig. 3:**
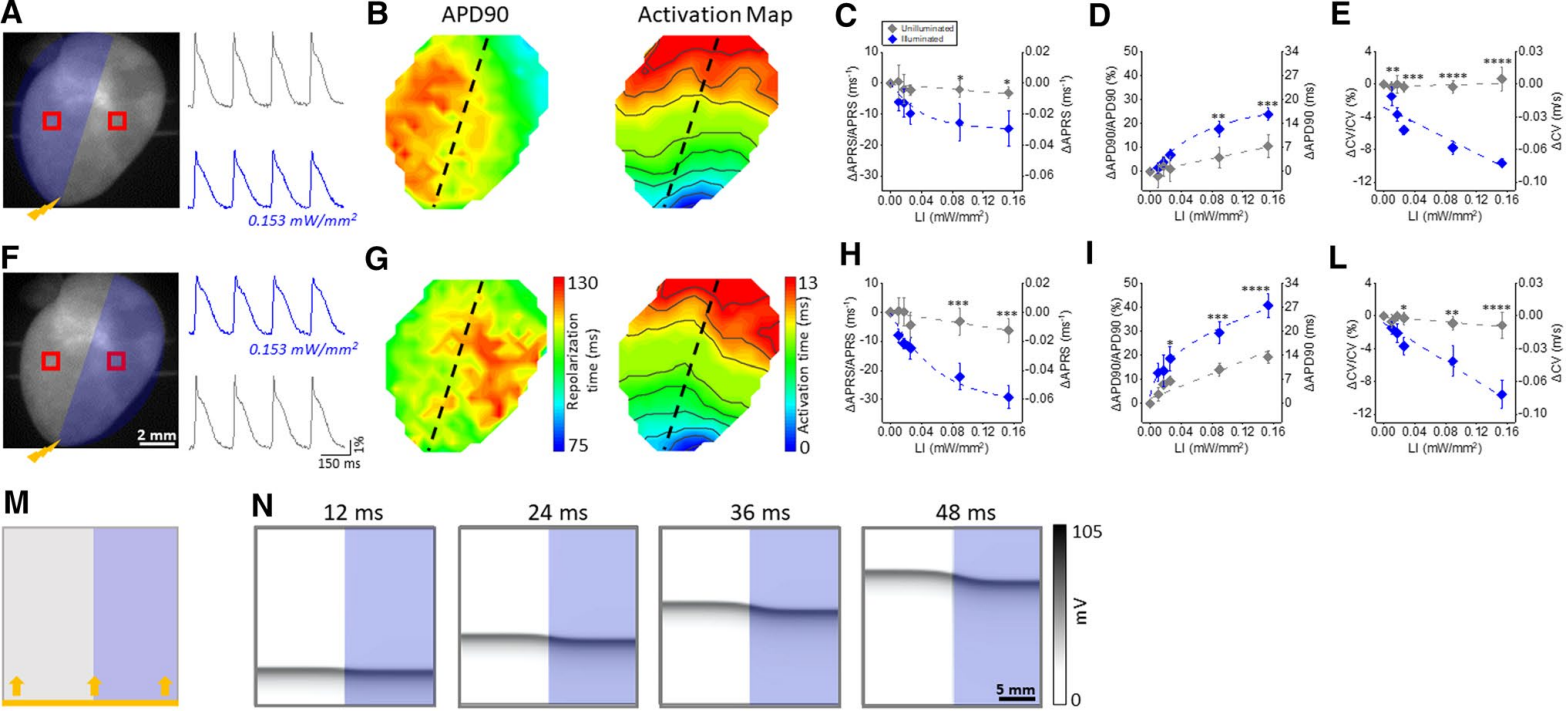
Patterned sub-threshold illumination in ChR2 intact mouse hearts. **A**, **F** Left panels: representative fluorescence images of a mouse heart (the same reported in Fig. 2C) showing the illumination protocols. ChR2 mouse hearts were electrically paced at the apex with an electrode at 5 Hz (yellow bolt symbol) and at the same time the left (**A**) and the right (**F**) half of the heart was illuminated with increasing sub-threshold LIs. Right panels: fluorescent signal (ΔF/F) extracted from the red ROIs in the illuminated region in blue (LI 0.153 mW/mm^2^) and in the unilluminated region in gray. **B**, **G** APD90 (left) and activation (right) maps corresponding to experimental condition in A,F. Black dashed line indicates the border between the illuminated and unilluminated region. Percentage variation and absolute variation of APRS **C**, **H**, APD90 (D,I), and CV (E,L) measured in the illuminated region and in the unilluminated region. Data were collected from 6 ChR2 hearts. Data is reported as mean ± SEM and exponential or linear fits on experimental data was superimposed. A two-way RM ANOVA with Tukey’s post hoc test was applied. M) Scheme showing the illumination protocol tested in a simulation of an optogenetically-modified mouse ventricular monolayer. The simulation domain (2.5 × 2.5 cm) consists of a mouse ventricular monolayer expressing ChR2 in which APs propagate as plane waves from the bottom (yellow arrows) to the top side of the domain. APs were electrically elicited with a stimulation frequency of 5 Hz. At the same time half of the domain was continuously sub-threshold illuminated (LI 0.02 mW/mm^2^) with the illumination edge perpendicular to the AP wave-front. N) Representative frames of the simulation movie showing a clear delay of the propagating AP wave in the illuminated region compared to the unilluminated region

To demonstrate the spatial-specific capability of sub-threshold illumination to manipulate electrophysiological parameters, we applied a simplified illumination pattern in ChR2 mouse hearts, where either the right or the left half of the ventricles was subjected to sub-threshold illumination (Fig. 3A, F). Traces in Fig. 3A, F clearly show light-induced changes of electrophysiological parameters in the illuminated region which were absent in the unilluminated half (LI 0.153 mW/mm^2^). As expected, APD90 and activation maps obtained in the presence of patterned sub-threshold illumination (Fig. 3B, G) show APD dispersion and wave-front distortions. We found that in the illuminated region APRS was reduced (Fig. 3C, H), APD90 was prolonged (Fig. 3D, I), and CV was reduced (Fig. 3E, L) as a function of irradiance, with differences achieving statistical significance at high LIs. Finally, a similar geometry was also applied in silico where half of the simulated domain was subjected to sub-threshold illumination (LI 0.02 mW/mm^2^), with the illumination edge perpendicular to the direction of wave propagation (Fig. 3M). As the representative frames in Fig. 3N show, waves propagating in the illuminated region exhibited a delay when compared to those propagating in the unilluminated region. This illumination pattern generated AP wave-front distortions as well as CV heterogeneities. These results confirmed those observed in our experiments: sub-threshold illumination with a simple geometry enables the generation of spatio-temporal heterogeneities in cardiac tissue, with regards to AP morphology and CV.

### Pacing rate dependency of sub-threshold illumination in intact mouse hearts

**Fig. 4:**
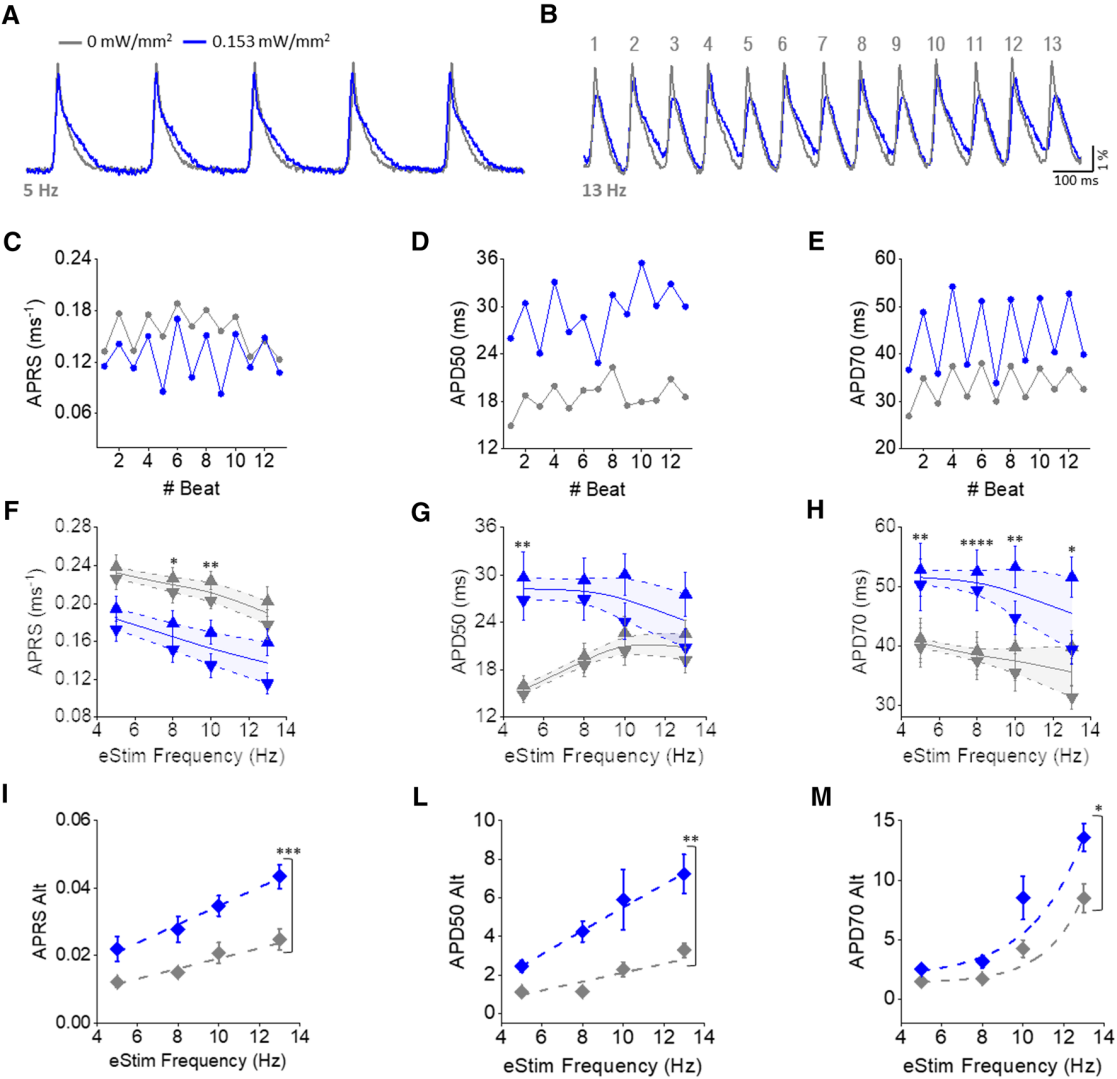
Pacing rate dependency of sub-threshold illumination in ChR2 intact mouse hearts. A, B) Representative traces optically recorded at the pacing rate of 5 Hz (**A**) and 13 Hz (**B**) during light-off (LI 0 mW/mm^2^, in gray) and light-on (LI = 0.153 mW/mm^2^, in blue) condition. **C**–**E** Graphic representation of APRS (**C**), APD50 (**D**) and APD70 (**E**) oscillations regarding the 13 APs showed in **B**. **F**–**H** APRS, APD50 and APD70 during odd (downward pointing triangles) and even (upward pointing triangles) beats. APRS, APD50 and APD70 mid-values are also shown (solid line) by averaging the even and odd beats. I-M) APRS, APD50, and APD70 alternans magnitude (Alt) measured during light-off and light-on condition. Data was collected from 8 ChR2 hearts. Data is reported as mean ± SEM and exponential or linear fits on experimental data was superimposed. A two-way RM ANOVA with Tukey’s post hoc test was applied to mid-values, while a two-way RM ANOVA with Tukey’s post hoc test was applied to alternans

Next, we investigated how the effects of sub-threshold illumination depend on the pacing rate. To this end, Langendorff-perfused mouse hearts expressing ChR2 were electrically paced at the apex with a burst of 15 stimuli with increasing stimulation frequency (5, 8, 10, 13 Hz). Cardiac activity was optically mapped in the presence (light-on) and absence (light-off) of sub-threshold illumination of the whole heart with LI 0.153 mW/mm^2^. Traces in Fig. 4A clearly show that at a low pacing rate (5 Hz), the sub-threshold illumination promoted changes in AP shape as previously observed. On the other hand, at a higher pacing rate (13 Hz in Fig. 4B), the beat-to-beat oscillations of APRS and APD were more pronounced when the sub-threshold illumination was applied (Fig. 4C–E). Based on this observation, we evaluated whether pacing rate impacts the effects of illumination in terms of beat-to-beat oscillations and mid-values (calculated by the average between the even and odd beats). We observed that the ChR2-mediated depolarizing current significantly increases APRS- and APD-alternans especially at high pacing rates (Fig. 4F–M). In terms of mid-values, we found that ChR2-mediated reduction of APRS (Fig. 4F) as well as APD70 prolongation (Fig. 4H) were preserved at each stimulation frequency, showing a negligible pacing rate dependency. In contrast, we found that APD50 was significantly prolonged only at slower pacing rates (Fig. 4G).

### Self-termination of electrically-stimulated VTs in ChR2 intact mouse hearts by sub-threshold illumination

**Fig. 5:**
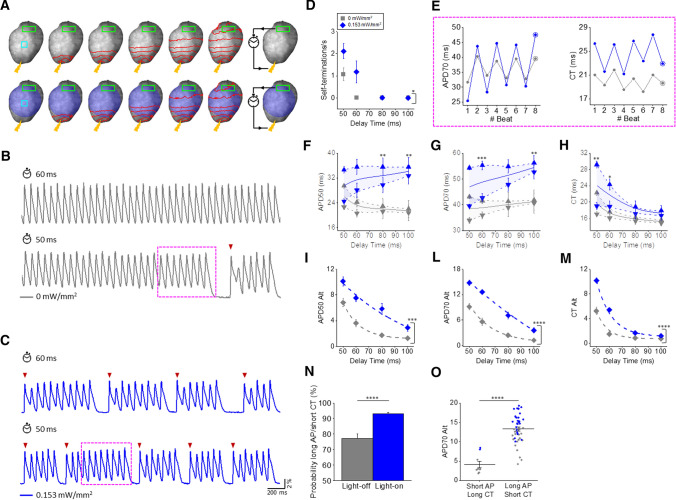
Sub-threshold illumination in ChR2 intact mouse hearts during electrically stimulated VT. **A** Scheme showing electrically stimulated VT in intact mouse heart expressing ChR2 in the presence (bottom) and absence (top) of sub-threshold illumination of the whole heart with LI = 0.153 mW/mm^2^. An electrical pulse (yellow bolt symbol) was applied to the apex of the ventricle to induce an AP which propagated towards the base of the heart (red dashed lines). Once the propagating wave was optically detected in the user-defined ROI (green rectangle), the system reinjects the electrical stimulus at the apex of the heart with a pre-defined fixed DT (clock). **B**, **C** Fluorescent signals (ΔF/F) extracted from the cyan ROIs showing VTs stimulated with a DT of 60 ms and 50 ms in the absence (traces in gray) and presence (traces in blue) of sub-threshold illumination. Spontaneous APs (red arrowhead) detected in the green ROI in **A** are needed to start or re-start the re-entrant cycle. **D** Number of interruptions per second of VT as a function of the DT, measured under light-off (LI = 0 mW/mm^2^, in gray) and light-on (LI = 0.153 mW/mm^2^, in blue) conditions. E) APD70 (left) and CT (right) oscillations in VTs showed in B and C (dotted magenta frame). The last beat before VT block has a long AP and a short CT. **F**–**H** APD50, APD70 and CT during odd (downward pointing triangles) and even (upward pointing triangles) beats. APD50, APD70 and CT mid-values are also shown (solid line) by averaging the even and odd beats. I-M) APD50, APD70 and CT alternans magnitude (Alt) measured under light-off and light-on conditions. A linear or an exponential fit was superimposed on experimental data. N) Probability that the last beat before VT block displays both a long AP and short CT calculated under light-off and light-on conditions. **O** APD70 Alt in several VT terminations measured under light-off and light-on conditions as a function of the last beat before VT termination with a short AP/long CT or a long AP/short AP. Data were collected from eight ChR2 hearts. Data are reported as mean ± SEM. Two-way ANOVA with Tukey test means comparison (**F**–**M**) and Student’s *t* test (**N**, **O**) were applied

With the aim of exploring the role of AP parameters alternans in the spontaneous termination of rapid rhythms, we used real-time feedback control to mimic re-entrant VTs in ChR2 intact mouse hearts. The system applies an electrical pulse to the apex of the heart, which induces an AP that propagates toward the base. Once the AP is optically detected at the base, the system reinjects an electrical stimulus at the apex with a pre-defined fixed delay time (DT: 50, 60, 80, 100 ms) to reinitiate the cycle (Fig. 5A, B). As expected, we found that by imposing long DTs the VTs were stable, but when reducing the DT VTs became unstable and prone to spontaneous termination (Fig. 5B). These dynamics can be explained by a loss of excitability as a consequence of rapid cardiac activity. In this respect we found that in 97 ± 1.9% of cases (tested in 8 ChR2 hearts), VT self-termination stems from the electrical stimulus failing to excite the apex because it falls within the refractory phase of the tissue. When this occurred, no propagated AP was optically detected at the base, thus interrupting the cycle. In such cases, activation of the detection site at the base by a sinus beat (red arrowhead in Fig. 5B) was required to re-start the VT. These dynamics were significantly altered during sub-threshold illumination (whole heart at LI = 0.153 mW/mm^2^). Representative traces in Fig. 5B, C show electrically stimulated VTs using a DT of 60 ms and 50 ms, in the absence and presence of sub-threshold illumination. With a DT of 60 ms, the VT was stable without illumination but displayed 4 episodes of self-termination over the same time period when the light was on. With a DT of 50 ms, 2 self-termination episodes occurred even without illumination, but their number increased to 5 over the same time period when the light was on. Overall, self-termination was not observed with DT > 60 ms; however, at DTs ≤ 60 ms the number of self-terminations was increased by illumination (Fig. 5D). This result suggests that one or more light-mediated effects may reduce the stability of VT by increasing the tendency to spontaneous termination. Focusing on the dynamics of representative traces in Fig. 5B, C (magenta dotted frame), we observed that the antiphase oscillations of APD and CT were more pronounced when sub-threshold illumination was applied (Fig. 5E). Importantly, the last beat before the spontaneous termination was characterized by a long AP and a short CT, eventually leading to VT block (circled point in Fig. 5E).

The question arises whether illumination facilitated VT self-termination by its effect on the mid-values of AP parameters or rather on the amplitude of their oscillations (alternans). To address this question, we compared the light-induced changes in the mid-values of individual AP parameters as a function of DT. APD was mainly prolonged by light at long DTs (Fig. 5F, G) when VT self-termination was seldom observed. CT was uniformly prolonged by light at all DTs, (Fig. 5H); however, longer CTs prolong the overall circuit time, which should favor perpetuation of the VT, as opposed to its self-termination. Thus, overall, light-induced changes in the mid-values of AP parameters may not account for VT self-termination. On the other hand, illumination enhanced both APD and CT alternans preferentially at short DTs (Fig. 5I–M), i.e., when VT self-terminations was more often observed. These results suggest an important involvement of cardiac alternans in self-termination of VTs. To quantify this effect, we calculated the probability of the long AP/short CT combination in the cycle preceding VT termination (circled symbols in Fig. 5E). During the light-off condition this probability was 77 ± 3% and it increased to 93 ± 1% (Fig. 5N) during illumination. This result suggests that light-mediated VT termination is primarily caused by electrical oscillations rather than by an overall change in AP parameters. Regardless of illumination state, in the cycle preceding VT self-termination the long AP/short CT combination was associated with a greater APD alternans amplitude compared to terminations preceded by a short AP/long CT (Fig. 5O). Finally, we investigated how the light-mediated current could affect the spatial distribution of electrical alternans across the ventricle by analysing APD50, APD90 and CT within 4 ROIs evenly distributed across the ventricle surface. We found that in all ChR2 hearts (*n* = 8), electrical alternans was spatially concordant both in the absence and presence of sub-threshold illumination and at all CLs.

## Discussion

In the present work, sub-threshold optogenetic stimulation was used to destabilize re-entrant arrhythmias in an experimental model system. ChR2 activation elicits an inward current proportional to the level of irradiance. Our experiments and simulations investigated the consequences of this effect at different scales, ranging from the single cardiomyocyte to the whole heart, by using transgenic mice constitutively expressing ChR2.

Illumination enabled manipulation of several electrophysiological parameters in ChR2 expressing cardiomyocytes. Light-induced effects were totally and immediately reversible. It’s important to stress that the light-induced effects were exclusively related to ChR2 activation; indeed, illumination did not impact electrical activity in cells from CTRL mice. Both at rest and in the presence of electrically stimulated APs, we observed gradual membrane depolarization, whose magnitude was a function of irradiance. Stable V_rest_ values were immediately established when constant illumination was applied, which is probably due to the gating properties of ChR2: at the onset of the light stimulus the channel opens rapidly, and then desensitizes to a smaller steady-state conductance during continued illumination [28]. Optogenetic illumination allowed us to obtain graded “sub-threshold” (i.e., that failed to trigger an AP) depolarization over a rather wide range of potentials (Fig. 1). This is likely due to the gradual increase of V_rest_ afforded by the protocol, which may inactivate Na^+^ channels before they enter an auto-regenerative loop with membrane potential. Membrane resistance (*R*_m_) at a diastolic potential did not vary substantially during sub-threshold illumination, albeit it tended to decrease when light was stitched on. At rest potential, membrane conductivity is too large to see any significant variations caused by the opening of a few ChR2 channels. A significant decrease of *R*_m_ by ChR2 opening could potentially be observed at more positive potentials, when inward rectified K^+^ current (I_K1_) is inactivated. However, AP plateau is very short in mouse cardiomyocytes, and measuring resistance during AP repolarization is therefore technically challenging. The light-activated *I*_ChR2_ also allowed AP manipulation in terms of amplitude and duration, in an irradiance-dependent manner. Indeed, upon increasing the irradiance, we observed a gradual reduction of APA and APRS. These effects can be explained by partial inactivation of Na^+^ channels, secondary to *V*_rest_ depolarization. Accordingly, a refractoriness prolongation was also observed. Sub-threshold illumination also caused an overall APD prolongation during both continuous illumination and during time-specific illumination, where the sub-threshold stimulus is applied exclusively during the repolarization phase of AP. This response is consistent with the I_ChR2_ reversal potential [27]. Mouse APs display a fast initial repolarization which rapidly brings the membrane potential below 0 mV, causing most of the repolarization phase to occur at membrane potentials below 0 mV. As *I*_ChR2_ is inward below 0 mV (i.e., during the repolarization phase of AP in mouse heart), it opposes the repolarizing K^+^ currents, thereby slowing down repolarization kinetics.

The effects of illumination in intact hearts expressing ChR2 were consistent with those observed in isolated myocytes. We found a gradual decrease of the APRS as a function of increased irradiance, which is consistent with the depression of the fast depolarization rate observed in isolated cardiomyocytes (Fig. 1E). Likewise, prolongation of APD was found in a light intensity-dependent manner, which is consistent with the single cell experiments as well as the computational study. Upon increasing irradiance, in intact hearts we also found a reduction in CV, likely the consequence of partial I_Na_ inactivation by *I*_ChR2_-induced *V*_rest_ depolarization [23]. Of note, in CTRL mice, a similar value of APD90 was found in patch-clamp and optical mapping recording, suggesting that the VSD and blebbistatin employed in Langendorff experiments do not significantly perturb the electrophysiology of the heart.

We also investigated how changes in cardiac electrical activity promoted by I_ChR2_ could be affected by the pacing rate. We found a strong rate dependency of APD50. In fact, during fast repolarization (AP phase1), higher pacing rates results in more time above the reversal potential of the ChR2 channel (≈ 0 mV) where I_ChR2_ is outward rather than inward, thus giving rise to a repolarizing current which counteracts AP prolongation [13]. More importantly, we found that sub-threshold illumination also affects the dynamics of cardiac electrical activity. The physiological beat-to-beat oscillations in APRS and APD, which usually occurs at fast beat rates, were increased by sub-threshold illumination and this effect was more pronounced at high pacing rates. This result may be related to the optogenetic manipulation of the electrical restitution curve (supplemental figure S1). Indeed, the introduction of the depolarizing current I_ChR2_ may result in a slower ion channel recovery from inactivation, eventually leading to an increase in the slope of the restitution curve which would cause an increase in cardiac alternans amplitude [29].

The pro-arrhythmic effect of electrical alternans has been extensively demonstrated in various cardiac preparations [43]. Paradoxically, electrical alternans has also been observed before spontaneous termination of re-entrant rhythms [15]. In a recent study, Biasci and coauthors developed a simplified mathematical model capable of reproducing the electrical dynamics occurring in re-entrant rhythms and demonstrated how alternans are involved in generating non-sustained bursting rhythms [1]. Briefly, when CT and APD oscillate between beats, a stimulus delivered after a beat with a short CT and long APD will encounter a much shorter recovery time than the preceding stimulus. Consequently, the termination of re-entry based arrhythmias occurs preferentially at the stimulus site following a beat with a short CT and a long duration.

Based on this theoretical study, here we employed sub-threshold illumination as a tool to increase cardiac alternans during an ongoing VT and we experimentally dissected the role of alternans in the context of self-termination of VT. In this respect, we exploited the capability of our optical platform to electrically generate re-entrant VTs in ChR2 mouse hearts using real-time feedback-control. Our platform allowed us to test the effects of sub-threshold illumination during an ongoing VT. In general, several approaches can be used to experimentally induced re-entrant VTs, including ischemia and reperfusion, catecholamine infusion, sodium pentobarbital or caffeine. However, these methods do not allow direct control of the re-entrant circuit length and sometimes are not sufficient to induce re-entrant arrhythmias due to the small size of the mouse heart [25]. In contrast, our strategy aims to generate user-defined re-entrant circuits with different cycle lengths (CLs) depending on the delay time (DT) of the electrical stimulus. We found a greater tendency for VTs to spontaneously terminate when sub-threshold illumination was applied, suggesting the presence of one or more light-induced mechanisms leading to spontaneous termination. We found that DTs at which illumination promotes VT self-termination are associated with larger light-induced enhancements of APD and CT alternans. The larger oscillations lead to a higher probability of VT termination, which consistently occurred following a beat with a short CT and long AP. More importantly, the relationship between electrical alternans magnitude and the probability of block associated with the combination of a short CT and a long AP was present regardless of illumination, suggesting that alternans may play a role in terminating re-entrant rhythms. Although the current work deals with VTs modeled by mono-dimensional re-entrant circuits, the same mechanism can in principle also occur in other much more complex systems (occurring in larger hearts, e.g., human) where other termination mechanisms (such as collision of multiple spiral waves) can concomitantly occur [2]. Future investigations should be focused on exploring the role of alternans in more complex geometries. In this respect, panoramic [33] or volumetric [37] imaging could provide a more comprehensive view of cardiac dynamics across the whole heart surface as well as within ventricle walls, expanding the epicardial observations reported in this study. Furthermore, investigating the involvement of calcium oscillations during the light-mediated increase of APD alternans is essential, especially considering that altered calcium homeostasis often underlies the development of AP alternans in (patho)physiological conditions [45].

In conclusion, our results support the idea that electrical alternans is the main mechanism for self-termination of re-entry related tachycardias. Pharmacological interventions aimed at increasing the likelihood of electrical alternans at high pacing rates, such as the use of class 1a Na^+^-channel blockers which typically reduce conduction velocity and increase APD and refractoriness in the human heart [22], may mimic the effects of sub-threshold illumination, thus explaining their efficacy in reducing the risk of sustained arrhythmias in patients.

## Supplementary Information

Below is the link to the electronic supplementary material.

Supplementary file1 (DOCX 882 kb)
