# Supplementary material for: Optogenetic manipulation of cardiac electrical dynamics using sub-threshold illumination: dissecting the role of cardiac alternans in terminating rapid rhythms
Source: Basic Res Cardiol. 2022 Apr 29;117(1):25. doi: 10.1007/s00395-022-00933-8 (PMC9054908; doi:10.1007/s00395-022-00933-8)
Supplement: Supplementary file 1 — Supplementary file1 (DOCX 882 kb) [file 395_2022_933_MOESM1_ESM.docx]

**Supplemental Figures**

**
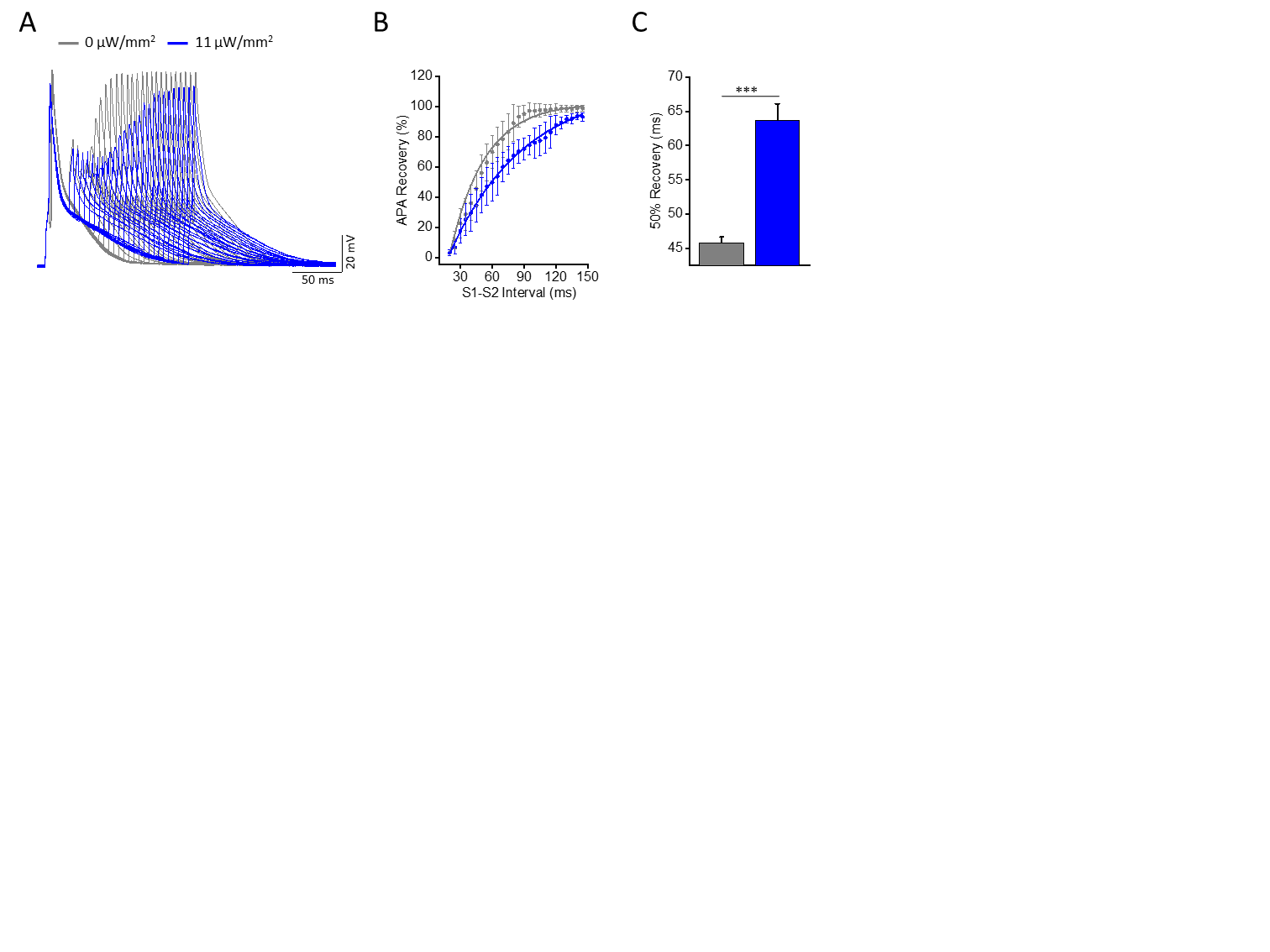
**

***Figure S1. APA restitution curve in ChR2 cardiomyocytes during sub-threshold illumination.*** *A) Representative traces of APs recorded with different S1–S2 intervals at baseline (in grey) and next from the same cell in the presence (11 µW/mm^2^, in blue) of sub-threshold illumination. Briefly, in this experiment, cardiomyocytes were electrically paced at 1 Hz. When the steady-state was reached, an extra-stimulus (S2) was introduced after the regular one (S1) and the protocol repeated at progressively longer S1-S2 intervals (20-145 ms). B) Restitution curves obtained by plotting APA recovery (%) versus the S1-S2 interval (ms) in the presence (in blue) and absence (in grey) of sub-threshold illumination. Data points were best fit to a single exponential with the equation y=a+b(1-exp(-x/c)). C) Values of 50% recovery of APA during S1-S2 protocol, in the presence (in blue) and absence of sub-threshold illumination. Data was collected from 4 ChR2 mice (6 cardiomyocytes). Data is reported as mean ± SEM and student’s t-test applied (***p<0.0001).*

*
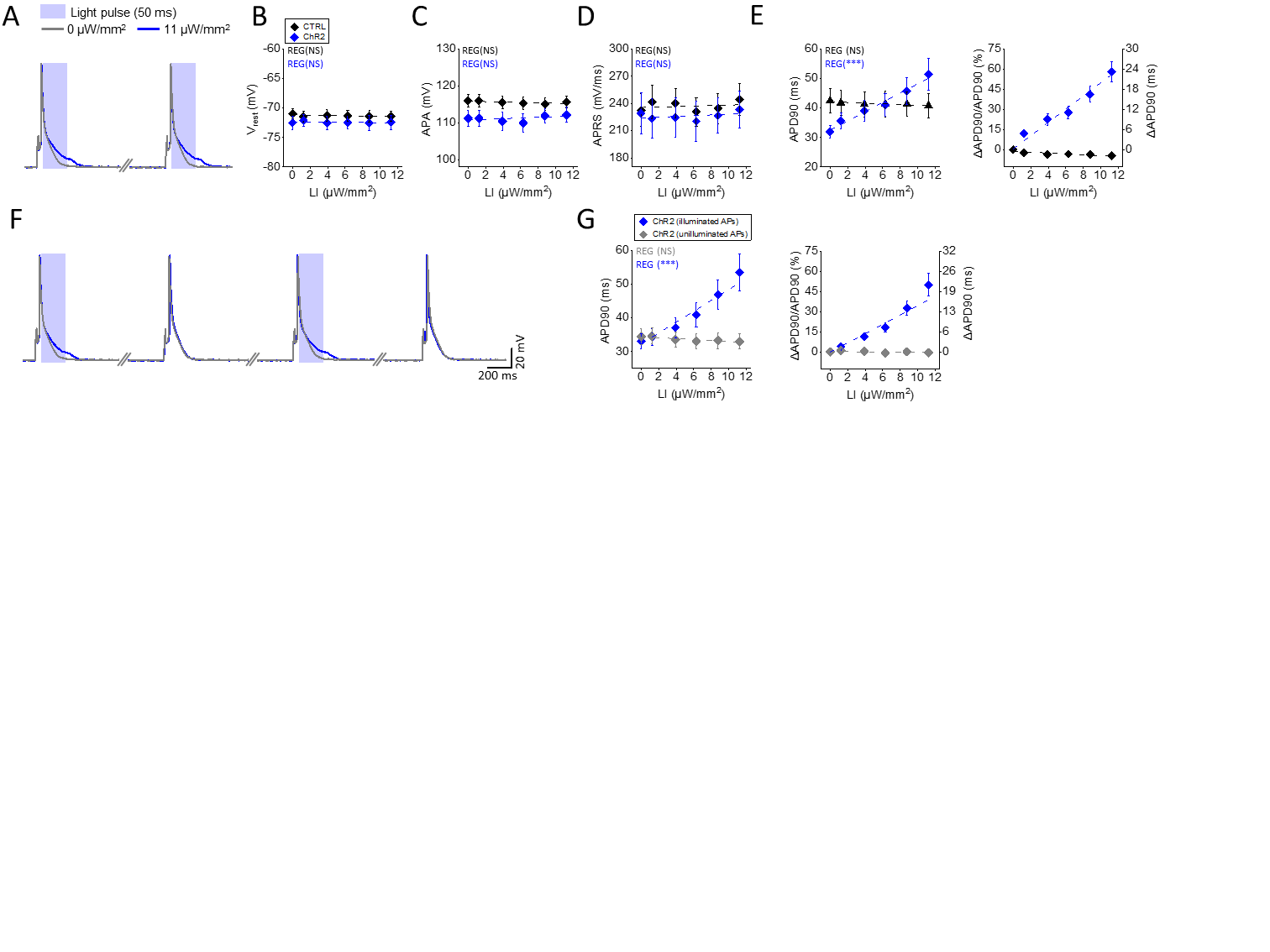
*

***Figure S2. Time-specific sub-threshold illumination in single cardiomyocytes isolated from CTRL and ChR2 mice.*** *A) Representative traces of APs recorded in a ChR2 cardiomyocyte in the absence (in grey) and presence (in blue) of sub-threshold illumination applied to all APs during repolarization phase. APs were induced by current injection at a frequency of 1 Hz. A light pulse (blue window, 50 ms duration, LI 11 µW/mm^2^) was delivered with a delay time of 10 ms after current injection. B-E) Absolute mean values of V_rest_, APA, APRS, APD90 and percentage variation and absolute variation of APD90 in CTRL (black diamonds) and ChR2 (blue diamonds) cardiomyocytes. F) Representative traces of APs recorded in ChR2 cardiomyocyte in the absence (in grey) and presence (in blue) of sub-threshold illumination applied in an alternating manner during AP repolarization phase. G) Absolute mean values (left), percentage variation and absolute variation (right) of APD90 in unilluminated (grey diamonds) and illuminated (blue diamonds) APs. Data was collected from 3 CTRL mice (20 cardiomyocytes) and 3 ChR2 mice (16 cardiomyocytes). Data is reported as mean ± SEM and a linear fit on experimental data was superimposed. Regression analysis results (REG; ANOVA test) are shown for both CTRL and ChR2 cardiomyocytes. No significant difference was found between CTRL and ChR2 cardiomyocytes in V_rest_, APA, APRS and APD in absence of sub-threshold illumination (two-way RM ANOVA test with Tukey’s post-hoc test).*

***
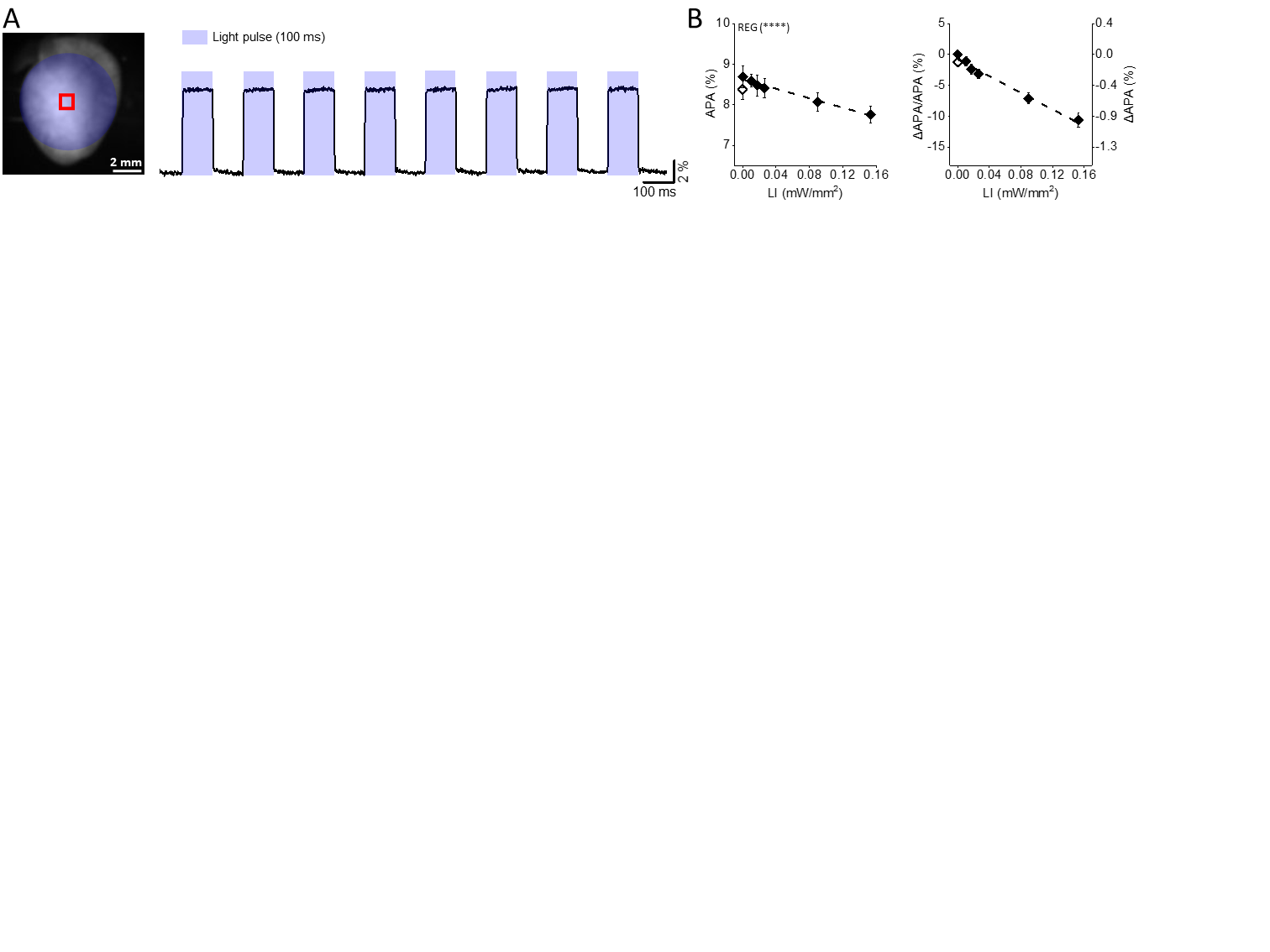
***

***Figure S3. Cross-talk between VSD and blue light impacts fluorescence signal during sub-threshold optogenetic illumination in CTRL mouse heart.*** *A) Left, representative fluorescence images (F0) of a CTRL mouse heart stained with a red-shifted VSD showing the illumination protocol. The entire ventricle surface of a CTRL mouse heart was sub-threshold illuminated with LI 0.153 mW/mm^2^, Light was applied in pulses of 100 ms duration, 5 Hz of frequency and in the absence of APs. Right, fluorescent signals (ΔF/F) extracted from the red ROIs. Blue rectangles show the light pulse delivered to the heart. B) Absolute mean values (left), percentage variation and absolute variation (right) of APA in CTRL hearts. Empty diamonds represent values when the light was turned off at the end of the illumination protocol to assess reversibility. Data was collected from 7 CTRL mouse hearts. Data is reported as mean ± SEM and a linear fit was superimposed. Regression analysis result (REG; ANOVA test) is shown.*

*
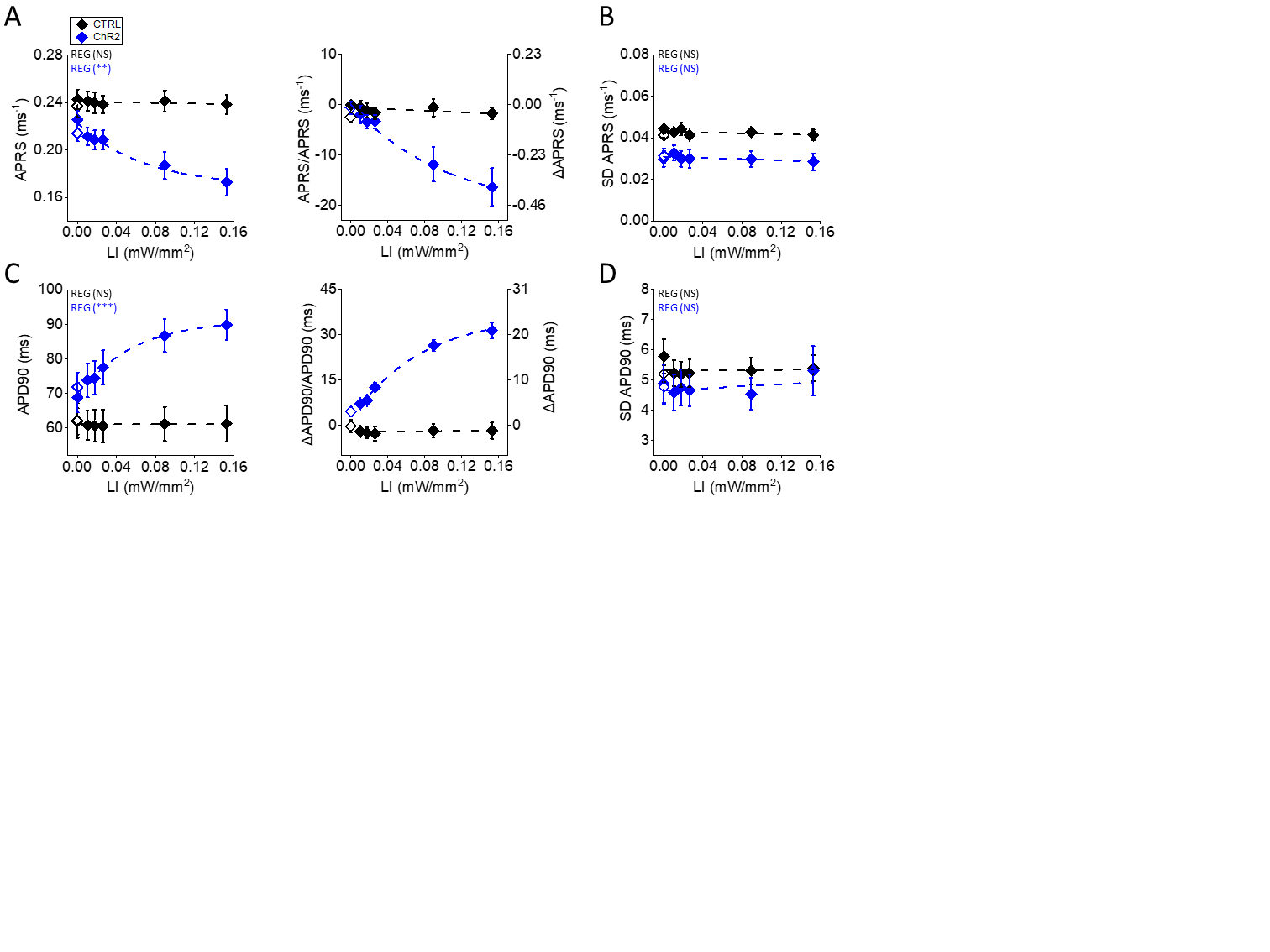
*

***Figure S4. APRS and APD90 analysis across the whole ventricle during sub-threshold illumination in CTRL and ChR2 intact mouse hearts.*** *APRS and APD90 analysis was performed across the whole ventricle using the corresponding maps. A, C) Absolute mean values (left), percentage variation and absolute variation (right) of APRS and APD90 in CTRL (black diamonds) and ChR2 (blue diamonds) hearts. B, D) Standard deviation (SD) of APRS and APD90 average across pixels values in CTRL (black diamonds) and ChR2 (blue diamonds) hearts. Empty diamonds represent values when the light was turned off at the end of the illumination protocol to assess reversibility. Data was collected from 7 CTRL and 7 ChR2 hearts. Data is reported as mean ± SEM and exponential (in A and C) and linear (in B) fit on experimental data was superimposed. Regression analysis results (REG; ANOVA test) are shown for both CTRL and ChR2 hearts. No significant difference was found between CTRL and ChR2 hearts in APRS, APD and CV in absence of sub-threshold illumination (two-way RM ANOVA test with Tukey’s post-hoc test).*

**
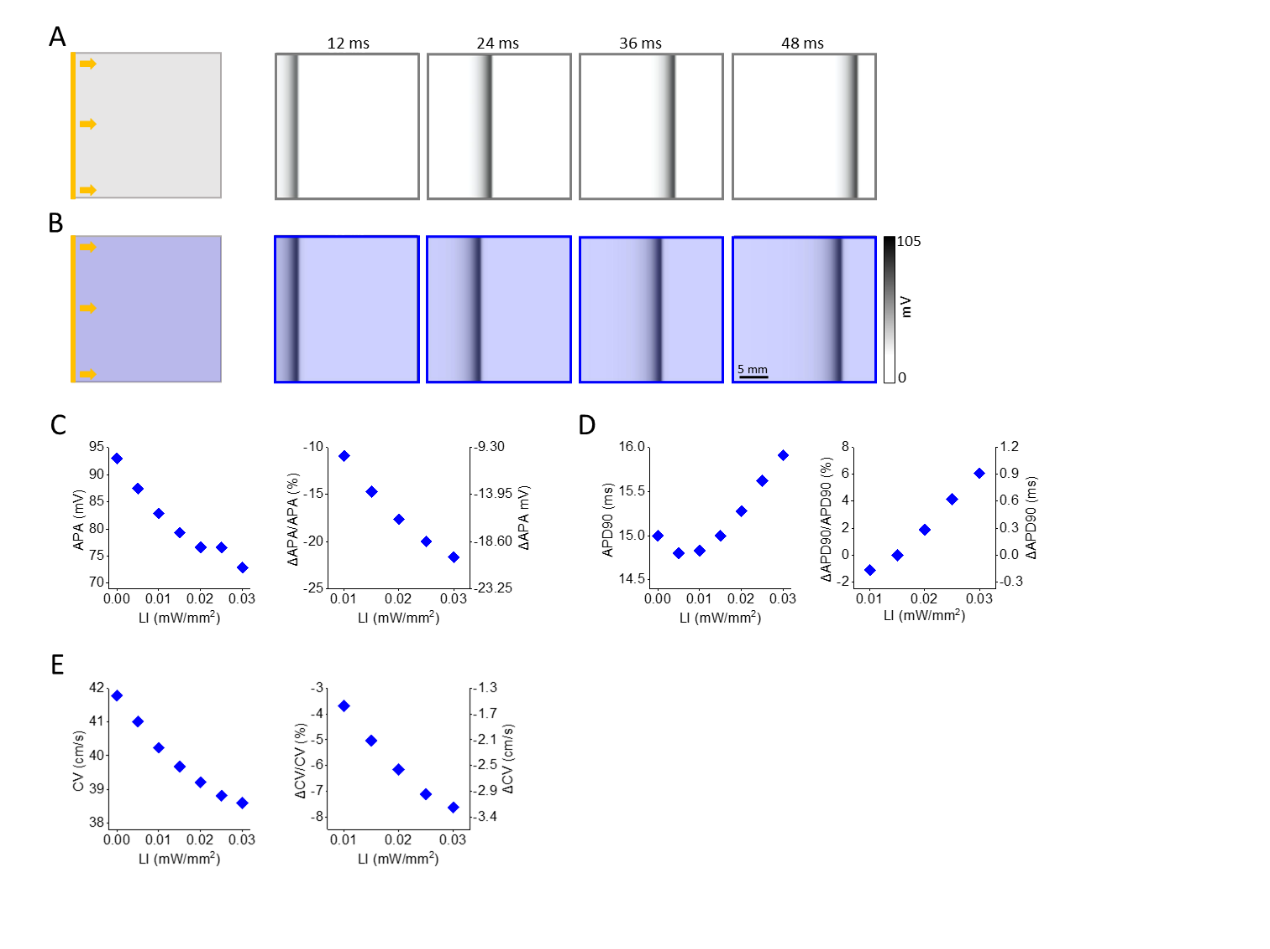
**

***Figure S5. Sub-threshold illumination in a 2D simulation of an optogenetically-modified mouse ventricular monolayer.*** *A-B) Left, scheme showing the simulation protocol. The simulation domain (2.5 × 2.5 cm^2^) consists of a mouse ventricular monolayer expressing ChR2 in which APs propagate as a plane wave from the left (yellow arrows) to the right side of the domain in the absence (A) and presence (B) of sub-threshold illumination of the domain with increasing LIs (0, 0.005, 0.01, 0.0153, 0.02, 0.025, 0.03 mW/mm^2^). APs were electrically elicited with a stimulation frequency of 5 Hz. Right panels: representative frames of the simulation movie showing AP propagates from the left to the right side of the domain with an isotropic CV of 0.42 m/s (A) and 38.6 cm/s (B) (LI 0.03 mW/mm^2^). C) D-E) Absolute values (left), percentage variation and absolute variation (right) of APA, APD (APD90) and CV measured in the domain.*
